# Castleman Disease Presenting as Renal Hilar Mass

**DOI:** 10.1089/cren.2015.0015

**Published:** 2015-11-01

**Authors:** Mohammad Hadi Radfar, Hamid Pakmanesh, Peyman Torbati

**Affiliations:** ^1^ Department of Urology, Labbafinejad Hospital, Shahid Beheshti University of Medical Sciences, Urology and Nephrology Research Center, Tehran, Iran.; ^2^ Department of Urology, Shahid Bahonar Hospital, Kerman University of Medical Sciences, Kerman, Iran.; ^3^ Department of Pathology, Labbafinejad Hospital, Shahid Beheshti University of Medical Sciences, Tehran, Iran.

## Abstract

***Background:*** We report a case of unicentric Castleman disease, a rare type of benign proliferation of lymphoid tissue. We present an uncommon disease that was managed effectively using laparoscopy.

***Case Presentation:*** A 32-year-old woman presented with left-sided flank pain. A large retroperitoneal mass was detected in the left renal hilum close to the renal vessels. Laparoscopic removal of the mass was effectively performed. The pathologic examination was in favor of a rare type of benign proliferation of lymphoid tissue compatible with Castleman disease. The patient was cured with no evidence of recurrence in 1-year follow-up.

***Conclusion:*** Transperitoneal laparoscopic approach is feasible and effective in the management of this disease and is curative.

## Introduction

Castleman disease is a rare type of benign proliferation of lymphoid tissue. Other nominations of this disease are giant or angiofollicular lymph node hyperplasia, lymphoid hamartoma, and angiofollicular lymph node hyperplasia. It is an uncommon lymphoproliferative disorder characterized by noncancerous growths (tumors) that may develop in the lymph node tissue at a single site or throughout the body.^1^ Unicentric Castleman disease involves tissue growth at only a single site. There are few or no symptoms other than those directly associated with the enlargement of the lymph node. Mass resection is almost always curative and no complementary treatment is recommended.^2^ The etiology is unknown and hyperproliferation of some B cells that often produce IL-6 is observed.^1^ In contrast, the multicentric disease is linked to HHV-8 and is associated with systemic presentations such as B-symptoms. No standard definitive treatment has been established for the multicentric disease.^2^

Involvement of the kidney is extremely rare in this disease.^3^ Williams and colleagues first reported laparoscopic removal of intraabdominal Castleman disease for the treatment of a subhepatic mass.^4^ This disease only rarely involves genitourinary tract.^5^ We report a case of unicentric Castleman disease, which presented as a retroperitoneal mass adjacent to the renal hilum.

## Case Presentation

**FIG. 1.**
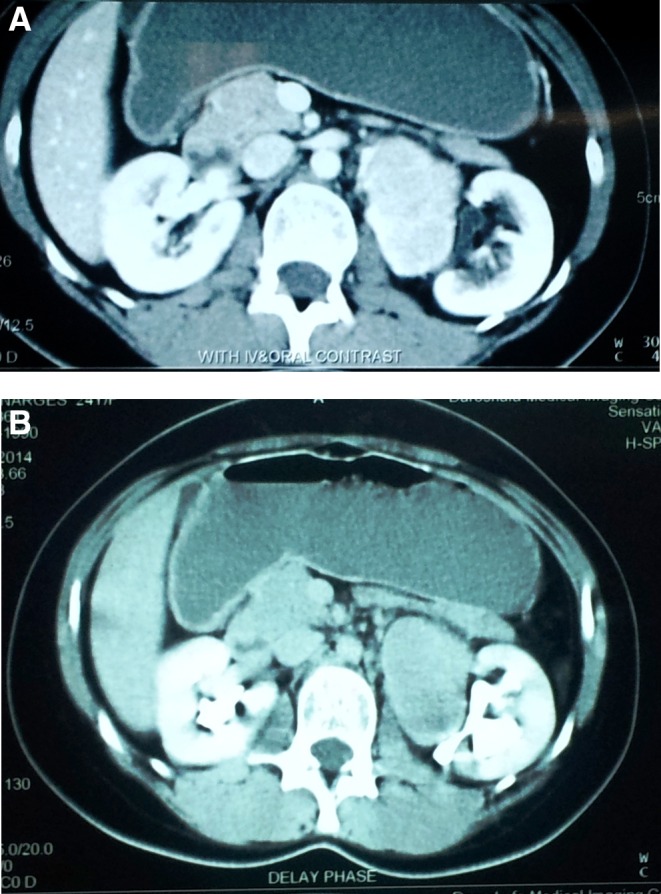
CT scan imaging revealed a mass in the left renal hilum that enhanced after injection of the intravenous contrast **(A)**. The lesion did not invade the pyelocalyceal system **(B)**.

A 32-year-old woman presented with dull left flank pain. Physical examination was normal. Abdominal ultrasonography revealed a solid mass with a diameter of 7 cm in the left renal hilum. Abdominal CT scan showed a single well-defined homogeneously enhanced mass close to the left renal pedicle with no calcification or necrosis (Fig. 1A, B). Laboratory data including catecholamine metabolites were in normal range. Chest and abdominopelvic CT scans were normal otherwise. The mass was resected completely laparoscopically using the transperitoneal approach. Although the mass was close to the renal vessels, there were no significant adhesions to the pelvis and to the renal parenchyma itself. The renal vessels were effectively shaved off the mass and the mass was resected with satisfactory margin. The mass was close to the renal vessels; however, the planes were distinguishable during laparoscopy and tumor was resected with acceptable margins.

**FIG. 2.**
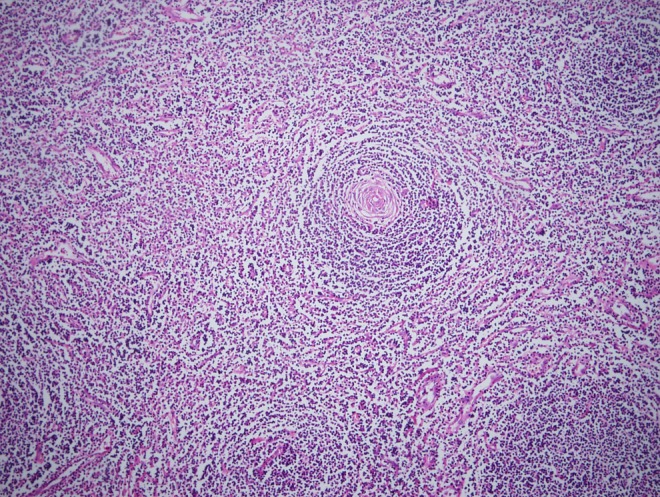
Pathologic examination of the specimen.

The pathologic examination was compatible with Castleman disease (Fig. 2). This woman underwent follow-up with no adjunctive therapy. No evidence of recurrence was present in the CT scan imaging at 1-year follow-up.

## Conclusion

Although Castleman disease is relatively rare, it should be one of the differential diagnoses in the management of masses with lymphoid proliferation. Transperitoneal laparoscopic approach is feasible and effective in the management of this disease and is curative.

## Abbreviations Used

CT: computed tomography
HHV-8: human herpes virus-8
IL-6: interleukin-6
